# Cystic Clear Cell Renal Cell Carcinoma: A Morphological and Molecular Reappraisal

**DOI:** 10.3390/cancers15133352

**Published:** 2023-06-26

**Authors:** Giacomo Maria Pini, Roberta Lucianò, Maurizio Colecchia

**Affiliations:** 1 Department of Pathology, IRCCS San Raffaele Scientific Institute, 20132 Milan, Italy; 2 IRCCS San Raffaele Scientific Institute, Vita-Salute San Raffaele University, 20132 Milan, Italy

**Keywords:** renal cell carcinoma, cystic renal neoplasm, differential diagnosis

## Abstract

**Simple Summary:**

Renal cancer is a common malignant neoplasm. Indeed, not every cancer is created equal, as there are different entities with specific morphological and molecular features. These differences also lead to different clinical behaviors, ranging from benign to highly aggressive neoplasms. In renal cancer, it is not unusual to have cystic hollow spaces. Clear cell renal cell carcinoma is the most frequent type of renal cancer, and it can be cystic. Distinguishing it from other subtypes of renal carcinomas can, in some cases, be challenging.

**Abstract:**

A wide variety of renal neoplasms can have cystic areas. These can occur for different reasons: some tumors have an intrinsic cystic architecture, while others exhibit pseudocystic degeneration of necrotic foci or they have cystically dilated renal tubules constrained by stromal neoplastic cells. Clear cell renal cell carcinoma (CCRCC), either solid or cystic, is the most frequent type of renal cancer. While pseudocysts are found in high-grade aggressive CCRCC, cystic growth is associated with low-grade indolent cases. The latter also form through a cyst-dependent molecular pathway, and they are more frequent in patients suffering from VHL disease. The differential diagnosis of multilocular cystic renal neoplasm of low malignant potential and clear cell papillary renal cell tumor can be especially hard and requires a focused macroscopical and microscopical pathological analysis. As every class of renal tumor includes cystic forms, knowledge of the criteria required for a differential diagnosis is mandatory.

## 1. Introduction

Renal cancer is a common malignant neoplasm whose classification has been expanding over the decades [1]. There are, indeed, more frequent and rarer subtypes of tumors, some of which are molecularly defined [2,3]. Morphological analysis is, however, still the basis of pathological diagnosis, and cystic areas can be present in a wide variety of renal neoplasms (whether benign or malignant) as minor or dominant components [4,5]. They are estimated to be present in 5–15% of lesions, where they reflect an inherent architecture of the tumor [4,6]. They must be distinguished from the pseudocystic degeneration of necrotic foci: while cystic growth is associated with a more indolent behavior, tumoral necrosis is present in aggressive masses [5]. This is especially true in cystic clear cell renal cell carcinoma (CCRCC), which is also the most frequent cystic renal cancer [4]. Cystic CCRCC is more frequent in patients with Von Hippel–Lindau (VHL) syndrome, and different molecular patterns are also implicated in its development compared with solid cases [4]. Nevertheless, cystic CCRCC is not classified as a separate pathologic entity. By contrast, multilocular cystic renal neoplasm of low malignant potential (MCNLMP) is independently identified in the WHO classification, despite molecular overlaps with CCRCC [5]. Cystic areas can be present in non-renal-cell neoplasms of the kidney as well, further complicating the diagnostic process [4]. In this review, we address the main morphological and molecular features of cystic CCRCC, together with its main differential diagnoses.

## 2. Macroscopic and Microscopic Features of Cystic CCRCC

According to the 2019 Bosniak classification (BC), the term “cystic renal mass” can be applied to neoplasms with a predominant cystic pattern and less than 25% enhancing tissue [7,8]. This term has an agnostic character, as it can imply both benign and malignant lesions [7,8]. A distinction must be made between renal cysts, which are benign, and solid neoplasms with minor cystic components. The latter are more likely to be malignant with pseudocystic degenerative areas with tumoral necrosis [7,8]. Both cystic growth pattern and pseudocystic degeneration can occur in CCRCC [6]. Less than 5% of CCRCCs have multiple cysts as their predominant architecture [4]. The minimum amount of cystic architecture necessary to define cystic CCRCC varies in the literature. Some authors mirror BC, as they require cystic areas of at least 75% [9], while others lower the threshold to 50% [10]. Interestingly, both cutoffs have proven to discriminate CCRCCs associated with a better prognosis [9,10].

Macroscopically, cystic growths appear as variably sized hollow spaces filled with clear or hemorrhagic fluid, with a clear separation from adjacent solid neoplastic tissue. Cysts can be single or multiple, with or without internal septations. When multiple cysts are predominant, the neoplasm can, overall, resemble a multilocular cyst. Evident solid areas have instead the typical golden-yellow color, with reddish hemorrhagic foci. Pseudocystic degenerative areas contain, instead, darker, denser, hemorrhagic material with cellular debris. They are more frequently centrally located within the lesion, and they can be surrounded by soft, greyish necrotic tissue. Vital parts of the tumor can also have, apart from the typical colors, whitish areas, where sarcomatoid differentiation is present.

**Figure 1:**
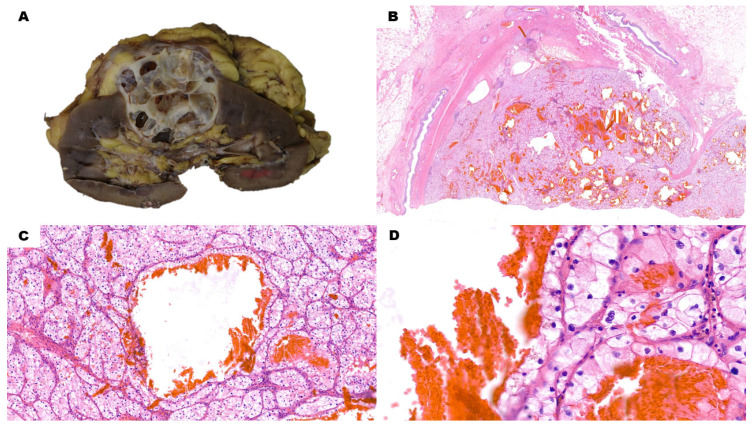
Cystic Clear Cell Renal Cell Carcinoma. (**A**) The gross specimen features a multiloculated, predominantly cystic nodule with variably thin walls and greyish solid areas. (**B**) (H&E) Low-power view of the lesion shows multiple, scattered, blood-filled cystic spaces, along with solid, whitish areas. (**C**) (H&E, 10×) Cysts are delimited by an epithelial lining with the same features of solid pericystic tissue. Around bigger cysts, higher magnification reveals the presence of microcystic spaces within neoplastic acini. No prominent nucleoli are evident at 10×. (**D**) (H&E, 40×) In this low-grade lesion, nucleoli are either very bland or absent, even with a high-power view.

Microscopically, along with macrocysts, even solid regions of CCRCCs can reveal a microcystic growth pattern (Figure 1A–D). Microcysts arise within tumoral nests, and cystic spaces are usually filled with red blood cells. Cells at the border of these microcysts do not have significantly different histological and immunohistochemical (IHC) features compared with solid acini. They have clear cytoplasm and variably sized nucleoli. Nuclei are usually basally located, although occasional apical alignment can be present. Positive labeling is present for carbonic anhydrase IX (CAIX) in a diffuse, box-shaped fashion, together with CD10, RCC, Vimentin and pan-cytokeratin. High-molecular-weight cytokeratins (HMWCKs) and CK7 are usually negative.

Microscopical analysis of cystic CCRCC usually reveals bland-looking clear cells with a low grade of differentiation (i.e., G1–G2 WHO grading). The epithelial coating of cysts, different from solid areas, is more likely to be CK7-positive, a feature that can be misleading in small biopsy samples. Nevertheless, HMWCKs are negative. In cases with a marked predominance of cystic growth, sampling of the capsule and septation can reveal clear cell clusters exceeding a 20× (1 mm) microscopic field, which is sufficient for a diagnosis of cystic CCRCC. Another criterion is the presence of an expansile growth of clear cells large enough to alter the contours of the capsule/septum. Finally, necrosis or vascular invasion could be present. Cellular clusters below the 20×/1 mm cutoff without expansile growth, necrosis or vascular invasion allow instead a diagnosis of MCNLMP [5].

**Figure 2:**
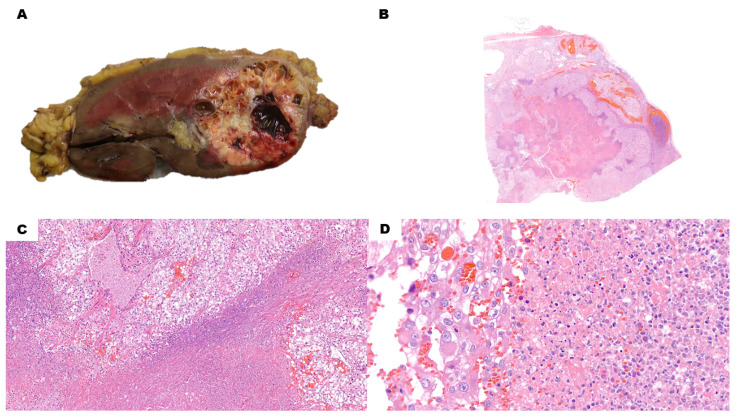
Pseudocystic Clear Cell Renal Cell Carcinoma. (**A**) Macroscopically, the nodule has a large hemorrhagic area surrounded by whitish and yellowish solid tissue. (**B**) (H&E) Low-power view shows a blood-filled area with blueish material at the border with the adjacent solid, whitish neoplastic tissue. (**C**) (H&E, 10×) The cystic area at the bottom of the picture, along with red blood cells, also contains blueish necrotic debris. Vital neoplastic cells at the top show prominent nucleoli already at this magnification, as it is a high-grade lesion. Other areas also showed rhabdoid cells. (**D**) (H&E, 40×) There is no clear-cut boundary between the necrotic debris on the right and vital solid neoplastic tissue on the left of the picture. This must be considered a pseudocyst rather than a cyst.

Pseudocystic degenerative areas are filled with nuclear and cytoplasmatic debris of necrotic cells, together with varying numbers of red blood cells (Figure 2A–D). No epithelial lining can be identified, and the surrounding tissue can be necrotic as well. Vital neoplastic cells are high-grade (i.e., G3–G4 WHO grading). Blandly eosinophilic cytoplasm and hyaline globules are commonly found in high-grade CCRCC, which can be misleading if clear cell areas cannot be identified. Moreover, CAIX tends to become positive near necrotic areas in different types of renal neoplasms as a hypoxia-induced factor [11,12]. As such, the diagnosis of high-grade pseudocystic CCRCC can be challenging and requires more extensive sampling.

## 3. Molecular Features of Cystic CCRCC

In CCRCC, tumor-initiating molecular alterations involve the deletion of the 3p chromosome [13]. Specifically, loss of the 3p25 region is observed in 85% of CCRCCs [14]. As the *Von Hippel–Lindau (VHL)* tumor-suppressor gene is located in this area of the DNA, 3p25 deletion leads to the loss of one allele. The second *VHL* allele is, instead, inactivated either by mutation or methylation. Mutations of *VHL* are found in 64% of CCRCCs [14]. VHL protein is implicated in different molecular mechanisms, including microtubular stabilization for cilia formation and inhibition of the alpha subunit of hypoxia-inducible factor (HIF) [15,16]. When *VHL* is mutationally inactivated, the accumulation of HIFα upregulates vascular endothelial growth factor (VEGF), inducing angiogenesis. After initiating factors, other molecular events drive tumoral evolution towards different neoplastic subtypes [13,17]. For example, *BAP1* and *PBRM1* are two evolution-driver onco-suppressor genes (also located on chromosome 3p) mutated in 13% and 36% of CCRCCs, respectively [14]. Their mutations are mutually exclusive, leading to CCRCCs with different features. *BAP1*-mutated CCRCC is a high-grade neoplasm with poor vascularization, including renal cell carcinoma with sarcomatoid and rhabdoid features [18,19,20]. In these cases, also, *CDKN2A* deletions and increased expression of *MYC* transcriptional programs can be present [18]. Moreover, *BAP1*-mutated CCRCCs can be composed of large tumoral cells with abundant cytoplasm and a papillary architecture (reminiscent of RCC with MITF-family rearrangement), along with IHC positivity for racemase/AMACR and CK7 [19]. In addition, a rich T lymphocyte infiltration can be present. Such an immune-inflamed phenotype is characterized by immune activation and increased cytotoxic immune infiltration with upregulation of antigen presentation machinery genes and PD-L1 expression [18]. Infiltrated tumors are also enriched for chromosomal losses of 9p21.3 [21]. *PBRM1*-mutated CCRCC is a low-grade neoplasm with high levels of angiogenesis and lower levels of inflammation. Novel mutations can also be acquired by neoplasms during therapy with small molecules, giving rise to acquired drug resistance [22].

Different molecular patterns seem to be implied in the formation of cystic CCRCC, for which a cyst-dependent CCRCC progression pathway has been identified [4]. As previously mentioned, VHL contributes to cilia formation through microtubule stabilization. Loss of VHL is followed by an aberrant orientation of newly formed microtubules, which, in turn, hinders ciliogenesis. Such an effect upregulates the cell cycle, since cells without cilia cannot rest in the G0 phase, as differentiated cells would do. Therefore, cilia can be considered tumor-suppressor organelles, and their absence promotes the transition towards malignancy [23]. Loss of cilia is also associated with cyst development caused by impaired cellular signaling [15]. This process happens both in sporadic cystic CCRCC, as well as in inherited diseases, such as polycystic kidney disease (PKD) and VHL disease (VHLd) [24,25]. PKD and VHL diseases are therefore both considered among so-called ciliopathies [26]. The latter is an autosomal-dominant tumor syndrome: patients suffering from it develop renal cysts and CCRCC in 60% and 30% of cases, respectively [5,27]. Renal cancer in VHLd has been reported as early as 16 years of age, with a mean age of 37 years [28]. Renal cysts in VHLd are also potential precursors of CCRCC, as their epithelial linings can demonstrate dysplastic areas as well as loss of the remaining *VHL* non-genetically mutated allele [4]. It follows that CCRCC in VHLd is often both cystic and bilateral. Interestingly, just as *VHL* is an early cancer-initiator gene that requires further downstream molecular events, cyst formation cannot rely on VHL deficiency alone [23,29]. A critical role is played by GSK3β, a protein kinase that regulates cell proliferation, microtubule assembly, stability and dynamics [15]. Combined loss of VHL and GSK3β disrupts ciliary maintenance, and it is considered a key player in the cyst-dependent CCRCC progression pathway. The role of GSK3β is, however, yet to be fully elucidated, as evidence has also shown higher levels of expression both in PKD and in some CCRCCs [30,31]. According to these studies, its inhibition might actually be therapeutically useful to hinder cystic expansion and the progression of both PKD and CCRCCs [30,31].

## 4. Differential Diagnosis of Cystic CCRCC

As already mentioned, the range of renal neoplasms with cystic areas is wide. It encompasses every WHO group of tumors of the kidney (i.e., renal cell, metanephric, mixed epithelial and stromal, mesenchymal, embryonal and germ-cell tumors), including frequent and rare, adult and pediatric, and inherited and sporadic forms [5]. Attention must therefore be paid to patient age and the bilaterality of lesions. Pathological analysis must focus on the cellular lining of cysts, as well as the pericystic stroma and possible solid areas which can be focal.

Cystic areas in frequent renal neoplasms, such as chromophobe carcinoma, papillary carcinoma and oncocytoma, are possible but rather unusual [5]. Although rarer, the main differential diagnosis for cystic-predominant CCRCC is MNCLMP. Since the vast majority of CCRCCs harbor the VHL mutation, 3p copy number loss or both, tumors with clear cell histology lacking these alterations can often be reclassified as different established or emerging entities [32]. However, in the case of MNCLMP, there are molecular overlaps with cystic CCRCC, including deletion of the 3p chromosome and similar mutated genes which are part of the cyst-dependent pathway [5,33]. For this reason, MNCLMP might be considered a subtype of CCRCC, at the most indolent end of the spectrum. Nevertheless, it also has distinct clinical, morphological and molecular features that allow a separate classification [5,33,34]. MNCLMP accounts for less than 5% of renal tumors. It is usually incidentally detected as a monolateral lesion in patients slightly younger than CCRCC patients (median age: 55 vs. 62). The macroscopic appearance is entirely composed of variably sized cysts with a small total diameter (usually pT1, i.e., ≤7 cm) [5]. Neither solid nodules nor necrotic foci can be present. Even microscopical necrosis is not accepted, together with rhabdoid/sarcomatoid differentiation, lymphovascular invasion, frequent mitoses or any atypical mitosis. The epithelial lining of the cysts features one to a few layers of clear cells. Nuclei are randomly distributed, without a predilection for the apical portion of cells, and they must be low-grade (G1–G2 WHO grading). The capsule and septa are fibrous, and they can include clusters of clear cells, but they must be small (i.e., <1 mm or <20× microscopic area). When diagnostic criteria are strictly applied, tumors identified as MNCLMPs have a benign clinical behavior [5]. IHC analysis is not of aid in differential diagnosis with respect to CCRCCs, as they have the same profile [35]. Apart from the molecular similarities between MNCLMP and CCRCC, the former has also been shown to have a lower frequency of mutations. Six genes have been found significantly more frequently mutated in cystic CCRCC: SETD2, GIGYF2, FGFR3, BCR, KMT2C and TSC2 [36]. These are potential candidate genes that could help to elucidate the mechanisms in the development and progression of CCRCC, as well as in the differential diagnosis with MNCLMP [36].

**Figure 3:**
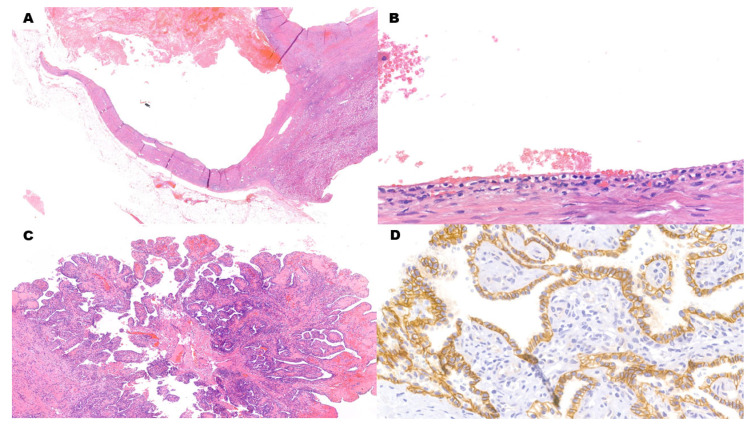
Clear Cell Papillary Renal Cell Tumor. (**A**) (H&E) Low-power view shows multiple blood-filled areas with fibrotic walls. (**B**) (H&E, 10×) The epithelial lining is composed of cuboidal to low-columnar clear cells. Nuclei in low-columnar cells tend to be oriented towards the cellular luminal apex. Nucleoli are not prominent. (**C**) (H&E) As the name of the tumor implies, papillary areas can also be present alongside cystic areas and they can protrude inside the cystic lumen. (**D**) (CAIX, 40×) Immunohistochemistry for CAIX signal has a cup-like pattern (i.e., with a missing luminal border). This pattern is typical of clear cell papillary renal cell tumors. The neoplasm is also CK7- and HMWCK-positive, while CD10 is negative.

Another benign renal cell tumor that can be nearly entirely cystic, featuring bland-looking clear cells, is clear cell papillary renal cell tumor (CCPRCT) (Figure 3A–D). Histologically, nuclei are oriented towards the luminal apex of the cells [37,38]. As in cystic CCRCC, CK7 is positive. However, CCPRCT also expresses HMWCKs (specifically, CK34βE12). CAIX signal has a cup-like pattern (i.e., with a missing luminal border), while CD10 is negative. Nevertheless, CCPRCT and low-grade CCRCC can have histologically identical areas, and unequivocal diagnosis of CCPRCT on needle biopsy may not be possible [5]. Molecularly, CCPRCTs have a distinct miRNA expression profile which also lacks the pattern typically associated with aggressive neoplastic behavior [39].

A cystic architecture combined with prominent nucleoli in epithelial cells can be found in WHO/ISUP category 5 neoplasms: tubulocystic RCC (TcRCC), acquired cystic disease-associated RCC (ACD-RCD), and eosinophilic solid and cystic RCC [2,5]. These neoplasms have a potentially misleading nucleolar appearance, as they look high-grade (equivalent to WHO grade 3) despite an indolent clinical behavior. They have eosinophilic cytoplasm, which distinguishes them from cystic CCRCCs. Moreover, TcRCC is composed of small cystic areas, which macroscopically reminds one of a sponge, rather than a multiloculated cyst. The cellular morphology ranges from flat to columnar, sometimes even with hobnail cells. Despite high-grade nucleoli, poorly differentiated or sarcomatoid areas must be absent, and the mitotic count is minimal. Differently from CCRCCs, CAIX is negative and racemase/AMACR is positive. ACD-RCDs are often multiple and bilateral solid masses in the setting of acquired cystic disease. As in VHLd, cysts are possible precursor lesions, and an ACD-RCD is often an intracystic mass. They are, however, derived from a history of long-term dialysis rather than an inherited gene mutation. Other than cystic areas, tubules lined by a multilayered epithelium with cytoplasmic vacuolation yield to a cribriform sieve-like pattern of growth. Other architectures might be present as well (e.g., papillary and solid). Oxalate crystals can be numerous within neoplastic tissue, and they are highlighted by polarized light. Both CD10 and racemase/AMACR are positive. In eosinophilic solid and cystic RCC, yellowish solid tissue is mixed with cystic spaces. Neoplastic cells are eosinophilic, but they also have basophilic intracytoplasmic inclusions surrounded by a clear halo (such inclusions are usually compared with Leishmania parasites). Binucleation and hobnail cellular profiles can also be present. Immunophenotypically, they are characterized by positive CD10 and racemase/AMACR, with a negative reaction for CAIX.

**Figure 4:**
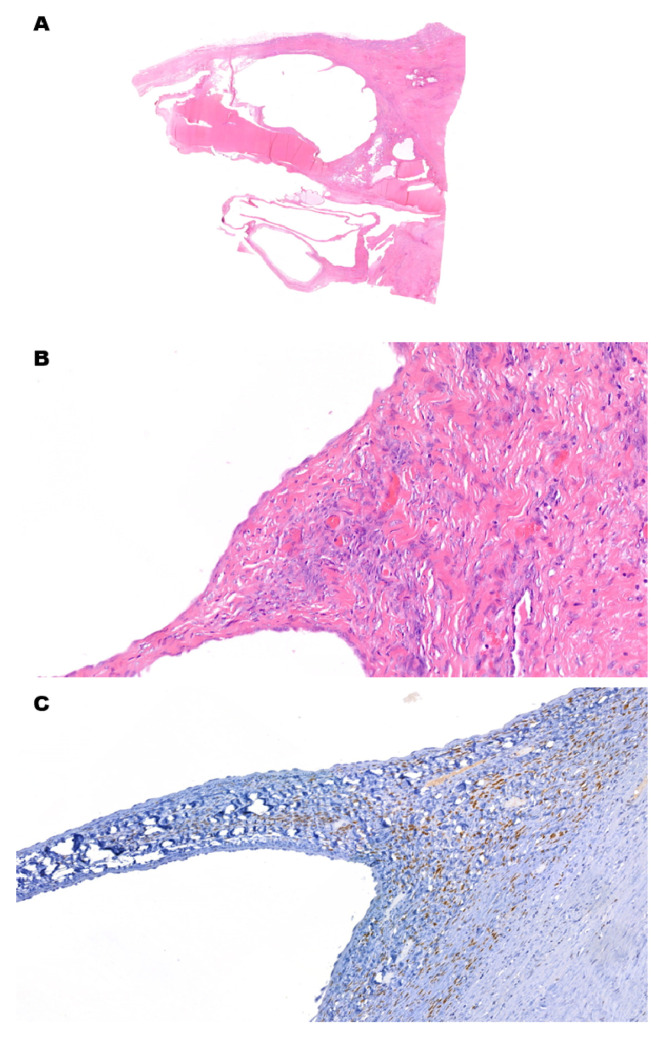
Mixed Epithelial and Stromal Tumor. (**A**) (H&E) Low-power view shows multiple empty cystic areas with fibrotic walls. (**B**) (H&E, 10×) The epithelial lining is flat and bland, while the pericystic stroma has foci with higher cellularity. Stromal cells are spindled and bland. These foci can be focal and hard to find. (**C**) (Estrogen Receptor, 40×) Immunohistochemistry for estrogen receptors is positive in stromal cells. They are also reactive for progesterone receptors and inhibin.

While cystic CCRCCs have fibrotic septa and capsules, other neoplasms are biphasic with specific stromal proliferations. Angiomyolipoma with epithelial cysts (AMLEC) is a rare subtype of angiomyolipoma, a benign mesenchymal tumor of the kidney which is part of the perivascular epithelioid cell (PEC)/PEComa tumor family. The majority of AMLECs are sporadic lesions, mainly occurring in middle-aged females, but some may be part of tuberous sclerosis. The latter may be suspected in young patients, with no sex predilection. Along with solid areas predominantly composed of smooth muscle and blood vessels, in AMLEC, cystic spaces are present. They have a cuboidal-to-hobnail epithelium and a dense pericystic stroma, similar to the cambium layer in rhabdomyosarcoma. The epithelium is cytokeratin-positive, while the cambium-like stroma and solid areas are cytokeratin-negative and positive for melanocytic markers (HMB-45, melan-A and MiTF). Adult cystic nephromas (ACNs) and mixed epithelial and stromal tumors (MESTs) are two other closely related biphasic neoplasms that usually arise in women [40,41,42]. Their biphasic nature is embodied by a renal cell epithelial component, along with the proliferation of bland-looking spindle stromal cells (Figure 4A–C). The morphology recalls ovarian stroma, together with the expression of estrogen and progesterone receptors, as well as inhibin. While ACN is entirely cystic, MEST has solid, whitish areas with different patterns of growth (e.g., glandular, papillary and thyroid-like). Pediatric cystic nephroma (PCN) is a similar lesion, epidemiologically restricted to children (usually males) below 2 years of age, and is molecularly characterized by a DICER1 mutation [43]. While it can be cured by radical excision, it can also be part of DICER1 syndrome. The latter is characterized by an increased risk of developing benign and malignant diseases, including Sertoli–Leydig cell tumors, pleuropulmonary blastoma and embryonal rhabdomyosarcoma. If any immature nephroblastic element is present in a PCN-like lesion, the diagnosis switches to cystic partially differentiated nephroblastoma (CPDN) [5]. While nephroblastoma has a slight female preponderance and is a malignant neoplasm, CPDN is more frequent among males, and it is cured by surgery in stage I disease. CPDN lacks solid nodules both on gross and microscopic examination. Cystic septa are lined hobnail epithelial cells, and the walls contain primitive WT1-positive blastemal cells that differentiate into abortive tubules or glomerulus-like structures.

Metanephric stromal tumor is another pediatric renal neoplasm which can have cystic areas. Solid parts show a concentric peritubular growth of spindle cells expressing CD34 and with BRAF v600e mutation [5,44,45]. While tubules are more commonly unaltered by the encircling spindle cells, some become obstructed and therefore cystically dilated, rendering a cystic gross appearance. This is a third mechanism of cystic formation, alongside the aforementioned cystic architectural growth and pseudocystic degeneration in necrotic areas.

Renal teratomas are rare, most often cystic and mature, with mixed epithelial and stromal elements [5,46]. They can be pure or accompanied by a yolk-sac component. Microscopically, cystic spaces can be lined by a keratinizing squamous epithelium with skin adnexa or, alternatively, by a thick fibromuscular stroma without any lining. Generally speaking, considering the rarity of primary renal teratomas, more frequent diseases must always be ruled out, including renal metastasis from a distant germ-cell tumor, direct extension from a retroperitoneal germ-cell tumor and teratoid nephroblastoma [5,46].

## 5. Conclusions and Future Directions

Our knowledge about CCRCC pathogenesis and its molecular features has been increasing over the years. This is important not only for identifying different subtypes of CCRCC, but it also allows us to track novel therapeutic targets and diagnostic markers. While there are molecular overlaps between cystic CCRCC and MCNLMP, there are also some significant differences. The validation of such data and the implementation of molecular studies in daily pathology practice will be of great aid in challenging differential diagnoses. Moreover, further clarifying the role of GSK3β in the formation and progression of cystic renal lesions could lead to a targetable protein in the treatment of cystic CCRCC, as well as PKD.

## Abbreviations

ACD-RCD	Acquired Cystic Disease-Associated Renal Cell Carcinoma
ACN	Adult Cystic Nephroma
AMLEC	Angiomyolipoma with Epithelial Cyst
BC	Bosniak Classification
CAIX	Carbonic Anhydrase IX
CCPRCT	Clear Cell Papillary Renal Cell Tumor
CCRCC	Clear Cell Renal Cell Carcinoma
CPDN	Cystic Partially Differentiated Nephroblastoma
HIF	Hypoxia-Inducible Factor
HMWCK	High-Molecular-Weight Cytokeratin
IHC	Immunohistochemical
MEST	Mixed Epithelial and Stromal Tumor
MiTF	Melanocyte-Inducing Transcription Factor
MCNLMP	Multilocular Cystic Renal Neoplasm of Low Malignant Potential
PEC	Perivascular Epithelioid Cell
PCN	Pediatric Cystic Nephroma
PKD	Polycystic Kidney Disease
TcRCC	Tubulo-Cystic Renal Cell Carcinoma
VEGF	Vascular Endothelial Growth Factor
VHL	Von Hippel–Lindau
VHLd	Von Hippel–Lindau disease
